# Effects of twin orientation and spacing on the mechanical properties of Cu nanowires

**DOI:** 10.1038/s41598-017-10934-6

**Published:** 2017-08-30

**Authors:** Zhenyu Yang, Lingli Zheng, Yonghai Yue, Zixing Lu

**Affiliations:** 1 Institute of Solid Mechanics, School of Aeronautic Science and Engineering, Beihang University (BUAA), Beijing, 100083 P.R. China; 2 School of Chemistry, Beihang University (BUAA), Beijing, 100083 P.R. China

## Abstract

The role of twin orientation in mechanical behaviors of nanomaterials is drawing increasing attention. In this paper, atomistic simulations on the tensile deformation of twinned Cu nanowires (NWs) are implemented to investigate the twin orientation and spacing effects. The results of numerical simulations reveal that the tensile deformation mechanisms can be divided into three types with the twin orientation varying from 0° to 90°: dislocations slip intersecting with twin boundary (TB), stacking faults formed parallel to the TB and TB migration. Detail analysis about dislocation motion is carried out to illustrate the plastic deformation mechanisms. In addition, with the increasing of the TB spacing, there is a transition from yield with strain hardening to yield with nearly constant flow stress. The peak stress decreases with the increase of TB spacing, which can be attributed to surface roughness caused by crystal reorientation. Our findings also suggest a possible approach to tune the mechanical behaviors of low dimensional nanostructures.

## Introduction

Twinned nanowires (NWs) have received tremendous attention owing to the superior mechanical performances. Experimental studies and molecular dynamics (MD) simulations have confirmed that nanomaterials containing nanoscale twins exhibit ultrahigh strength^1–4^ or even beyond the elastic limit^5^, significant strain hardening effects^6^ and crack resistance^7^. Based on the strengthening mechanism, the introduction of coherent twin boundaries (CTBs) has become a well-known means to strengthen nanocrystalline materials^8–11^. Previous researches mainly focused on the effects of TB spacing^6, 12, 13^ and cross-section shape^14^ on the mechanical properties of twinned NWs with the CTBs perpendicular to the NW axis. Recently, the significant influence of the twin orientation on the mechanical behaviors of NWs provoked more and more concern about the NWs with tilted twins. Twinned NWs with CTBs at a specific angle were presented to investigate the anisotropic size effect in strength^15^ and the deformation mechanism of three-point bending^16^. The mechanisms of TB migration^17, 18^ and shape memory effects^19^ were disclosed in nanomaterials with slanted twins, and a large amount of plasticity of the NWs could be achieved via TB migration.

Using finite element method, the effect of the angle between loading axis and CTBs on the deformation behaviors of nanotwinned Cu was studied^20, 21^. Numerical simulation results showed that a desirable combination of high tensile strength and ductility can be achieved by proper arrangement of the TB angle. In addition, comprehensive atomistic simulation studies were carried out on twinned Cu nanofilms and two predominant slip mechanisms, slip intersecting with the CTBs and slip parallel to the CTBs, were found to be responsible for the brittle-to-ductile transition with the changes of twin orientation^22^. However, a systematic research on the effects of twin orientation and especially the TB spacing on the mechanical properties of twinned NWs was seldom involved so far. In this paper, using MD simulations, we investigate the plastic deformation mechanisms of twinned Cu NWs with twin orientation changing from 0° to 90°. In addition, Cu NWs with twin orientation close to 30° have been prepared in experiment^23^, therefore Cu NW with titled twins with the angle of 35.26° is picked as a representative to study the size dependence of mechanical behaviors on TB spacing.

## Results

### Tensile deformation of Cu NWs with twin orientation of 0°, 70.53° and 90°

**Figure 1:**
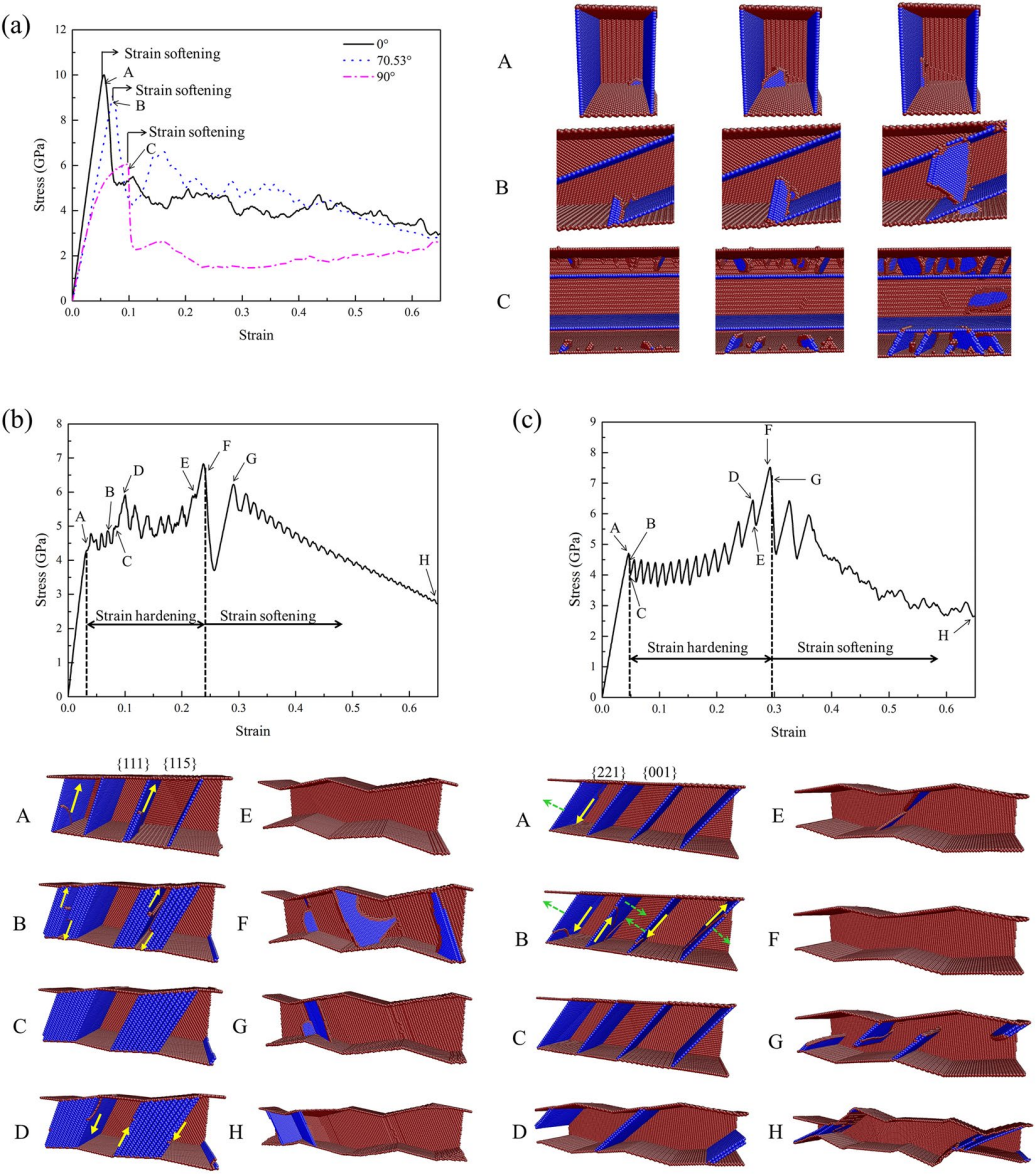
Tensile stress-strain curves and plastic deformations for Cu NWs with titled twins. **(a)** 0°, 70.53° and 90° samples. **(b)** 19.47° sample. **(c)** 35.26° sample. Yellow arrows and green dashed arrows denote the direction of dislocations slip and TB migration, respectively. The front surface atoms and the perfect FCC atoms are removed for clarity.

The typical tensile stress-strain curves for the twinned Cu NWs with the orientation of 0°, 70.53° and 90° are illustrated in Fig. 1(a). Each twinned NW has a 10 nm × 10 nm square cross-section and the TB spacing is about 6 nm (Supplementary Fig. S1). For those NWs, the stress drops sharply beyond the yield point with no strain hardening phenomenon, which is in agreement with what Sun *et al*. observed in nanotwinned Cu films^22^. It’s worth noting that there is discrepancy in Young’s modulus for various inclination angles on account of different lattice orientations (see Supplementary Table S1). Moreover, NW with CTBs perpendicular to the loading axis (*θ* = 0°) exhibits highest stress limit. The snapshots of defects evolution of Cu NWs around the initial yield point are also shown on the right side for the purpose of revealing the incipient plastic deformation mechanisms. According to Fig. 1(a), dislocations slip in the planes inclined to the CTBs at the initial yield point for 0°, 70.53° and 90° samples. For these samples, the resolved shear stresses within the planes parallel to the CTBs are very small even zero, preventing the dislocations motion in such planes. Slip intersecting with the CTBs leads to high peak stress without strain hardening, which was also reported in nano-twinned Cu films^22^.

### Tensile deformation of Cu NW with twin orientation of 19.47°

The tensile stress-strain curve and snapshots of the corresponding plastic deformation for the NW with twin orientation of 19.47° are shown in Fig. 1(b). The stress-strain behavior is apparently different from that of 0°, 70.53° and 90° samples. The stress-strain curve can be segmented into three stages: elastic deformation, strain hardening and strain softening. Dislocation slipping in the planes parallel to the CTBs dominates the plastic deformation process, with a large amount of stacking faults formed, as shown in Fig. 1(b). Leading partial dislocations with Burgers’ vector of 1/6〈112〉 emit from the junctions of the CTB-{115} free surface where stress concentration usually occurred^24^, leaving stacking faults behind (point A). Partial dislocations slip continuously in {111} planes parallel to the CTBs with the increase of strain (point B). When the tensile strain reaches 8.65%, the grains with {115} free surface are filled with stacking faults (point C). From then on, the NW undergoes elastic stretching until trailing partial dislocations emit subsequently at the strain of 10.08%, accompanied by the disappearance of stacking faults (point D). The twinned NW transforms into a single crystalline NW without residual defects at the strain of 22.51% and the NW starts to undergo another stage of elastic stretching, which finishes at the end of point E. This phenomenon is referred to the dislocation starvation, which is also found in tensile loading of single crystalline metal nanowires^25, 26^. Dislocations slip in different slip planes is observed beyond the peak stress at the strain of 24.11% (point F). Shortly after the abrupt stress drop, the defect-free NW is in another state of elastic elongation. Intriguingly, the stress decreases almost linearly with smaller amplitude with the further increasing of strain. The atomistic configurations at point G and H confirm that the abnormal occurrence of the limited dislocation activities in the only two slip planes.

### Tensile deformation of Cu NWs with twin orientation of 35.26° and 54.74°

Strain hardening is also observed in the tensile deformation of twinned Cu NWs with the orientation of 35.26° and 54.74°. For these NWs, partial dislocations slipping within twin planes, which results in TB migration, plays an important role in the process of strain hardening. Figure 1(c) indicates that the initial yielding of 35.26° sample begins with twinning partial dislocations nucleation from the junctions of the CTB-free surface at the strain of 4.60% (point A). Partial dislocations propagate on the twin planes with Burgers’ vector of 1/6〈112〉 (point B). Two dislocations slip upward and the others slip downward, as shown in Fig. 1(c). Partial dislocations annihilate at the free surface with CTBs moving a distance of one atomic layer at the strain of 5.02% (point C). The above-mentioned processes are repeated continuously until the two neighboring CTBs turn into stacking faults and the grains with {001} surface vanish (point D). As the tensile loading continues, the stacking faults annihilate by trailing dislocations nucleation and slipping cross the section of the NW, and then the initial twinned NW transforms into a defect-free single crystalline NW (point E to F). New dislocations nucleate from the junctions of {221} surface and the newly formed free surface beyond the peak stress at the strain of 29.56% (point G). The atomistic configuration at point H indicates that the final failure of 35.26° sample is expressed as sliding, which is also found in an *in-situ* tensile test of Cu NW with slanted twins^23^. For NWs with the twin orientation of 54.74°, the deformation process is similar. The only difference is that 54.74° sample fails to transform into a defect-free single crystalline NW. And the new dislocations slip intersecting with stacking faults retained in the NW, which results in the abrupt stress drop (see Supplementary Fig. S2). When TB spacing is reduced to 1.7 nm, the stacking faults formed by TB migration in 54.74° sample also have completely disappeared before the second yield point (see Supplementary Fig. S3). This size-dependent deformation behavior is closely related to surface roughness which will be discussed in detail upon the effect of TB spacing in 35.26° sample.

It needs to be emphasized that TB migration is a fast process, and the strain associated with CTBs moving a distance of one atomic layer is only about 0.4%, as shown in Fig. 1(c). The stress-strain curve of strain-hardening regime exhibits marked serration which is related to the discontinuous process of TB migration. Partial dislocations nucleation and slipping result in stress decrease. On the other hand, the defect-free twinned NW after each TB migration forms an intermediate phase, which is associated with the linearly ascent stages of stress-strain curve. Moreover, TB migration shows favored directionality with the elimination of grains with {001} and {114} free surface in 35.26° and 54.74° samples, respectively. The directionality of TB migration is further explained in detail below.

### Brief summary of tensile deformation mechanisms for Cu NWs with titled twins

As described in the previous sections, there are three kinds of plastic deformation modes for nanotwinned Cu NWs with the twin orientation ranging from 0° to 90°: (1) dislocations slip intersecting with the CTBs (0°, 70.53° and 90° samples); (2) partial dislocations slip within the planes parallel to the CTBs with stacking faults left behind (19.47° sample); (3) partial dislocations slip within the twin planes, resulting in TB migration (also known as detwinning) (35.26° and 54.74° samples). According to the yielding mechanisms, the NWs could be classified into two categories: strain softening (0°, 70.53° and 90° samples) and strain hardening (19.47°, 35.26° and 54.74° samples), as summarized in Table 1. The processes of formation and annihilation of stacking faults in 19.47° sample and TB migration in 35.26° and 54.74° samples are associated with the phase of slow strain hardening over a wide range of strain (more than 20%). The highest peak stress is achieved in 0° sample, and partial dislocations generating stacking faults are prevalent in NWs with small TB orientation angles, which is consistent with experimental observation in nanometals^27^.

**Table 1:** Summary of tensile deformation mechanisms for Cu NWs with titled twins.

Slant angle	Schmid factors	Dislocation activities after initial yield	Yielding mechanisms	Initial yield strain	Range of strain hardening	Peak stress (GPa)
0°	0	Slip intersecting with CTBs	Strain softening	5.60%	—	10.01
19.47°	0.27	Stacking faults	Strain hardening	3.15%	20.65%	6.83
35.26°	0.41	TB migration	Strain hardening	4.60%	24.64%	7.52
54.74°	0.41	TB migration	Strain hardening	4.71%	22.92%	8.41
70.53°	0.27	Slip intersecting with CTBs	Strain softening	7.20%	—	9.09
90°	0	Slip intersecting with CTBs	Strain softening	9.86%	—	6.06

The maximum Schmid factors for dislocations slip within the planes parallel to the CTBs are calculated in order to rationalize the different deformation mechanisms, as shown in Table 1. For the cases that the CTBs are perpendicular or parallel to the loading axis, the Schmid factors for the slip planes parallel to CTBs are zero (so-called hard orientation), disabling the dislocations motion within such planes. For the NWs with twin orientation *θ* close to 45°, the maximum Schmid factors for dislocations slip within twin planes are large enough to activate TB migration (so-called soft orientation). If the twin orientation is intermediate (19.47° and 70.53° samples), the Schmid factor is not the only reason for favored deformation mechanism^28^. Other factors such as free surface and axial orientation are probably responsible for the different plastic deformation modes of 19.47° and 70.53° samples.

### Effect of TB spacing in 35.26° sample

**Figure 2:**
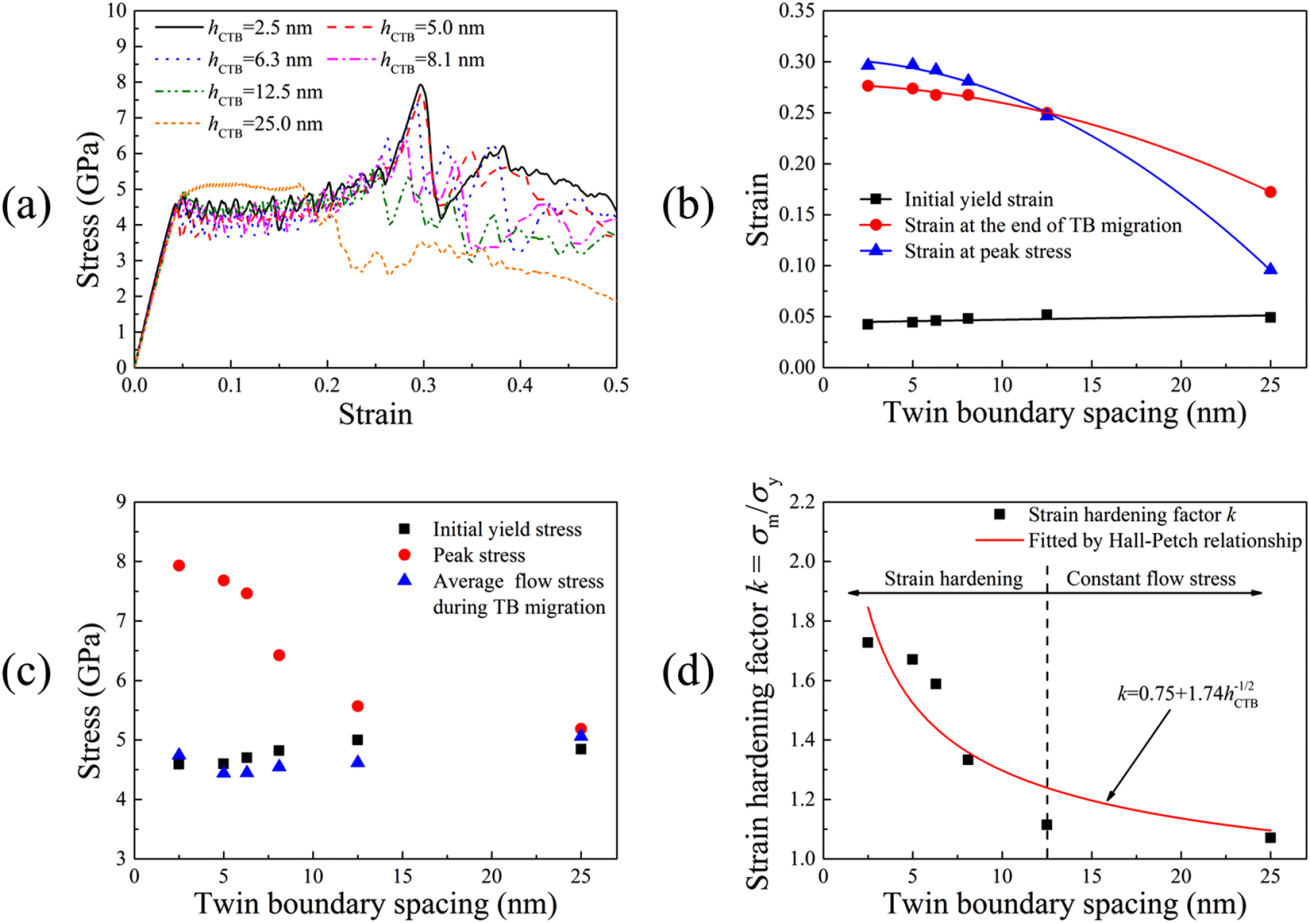
(**a**) Tensile stress-strain curves for 35.26° samples with TB spacing 2.5–25.0 nm. **(b)** Initial yield strain, strain at the end of TB migration and strain at peak stress versus TB spacing. **(c)** Initial yield stress, peak stress and average flow stress during TB migration versus TB spacing. **(d)** Strain hardening factor *k* = *σ*_*m*_/*σ*_*y*_ versus TB spacing. *σ*_*m*_ and *σ*_*y*_ denote the peak stress and the initial yield stress, respectively.

In this section, Cu NW with twin orientation of 35.26° is taken as a representative to investigate the effect of TB spacing (denoted as *h*_CTB_) on the tensile deformation behaviors. Figure 2(a) shows the tensile stress-strain curves for 35.26° samples with various TB spacing. It is clearly seen that each NW exhibits nearly constant flow stress in the strain range of 5~16%, which is induced by TB migration. Nevertheless, the strain hardening effect is not obvious in the samples with larger TB spacing (*h*_CTB_ > 10 nm). Young’s modulus calculated from the elastic deformation stage is about 107 GPa and the difference among the six samples is negligible. NWs with titled twins show independence of Young’s modulus on TB spacing, which is consistent with the results of NWs whose loading axis is perpendicular to CTBs^3, 6^. As presented in Fig. 2(b), the initial yield strain remains almost constant in spite of TB spacing change. The strain at the end of TB migration and the strain at the peak stress point decrease with the increase of TB spacing. It needs to be emphasized that the two samples with TB spacing *h*_CTB_ = 12.5 nm and 25.0 nm fail to transform into single crystalline NWs, and the stress almost keeps constant during plastic deformation stage (Fig. 2(a)).

With the average flow stress determined by averaging the stress between the initial yield strain and the strain at the end of TB migration, Fig. 2(c) illustrates the relationships between the initial yield stress, the peak stress, the average flow stress and the TB spacing. The peak stress (the maximum flow stress) decreases prominently with the increase of TB spacing. As previously stated, there are two yielding mechanisms for NWs with different TB spacing: yield with strain hardening and yield with nearly constant flow stress. Here, we use the strain hardening factor *k* = *σ*_*m*_/*σ*_*y*_ to characterize the strengthening effect, where *σ*_*m*_ and *σ*_*y*_ denote the peak stress and the initial yield stress, respectively. As shown in Fig. 2(d), the strain hardening factor *k* decreases with the increase of TB spacing but keeps constant when TB spacing is larger than 12.5 nm, which can be fitted by Hall-Petch relationship^29, 30^. There is a dividing line at the critical TB spacing *h*_CTB_ = 12.5 nm. NWs with TB spacing smaller than 12.5 nm exhibit obvious strengthening effect with strain hardening factor *k* much larger than 1.0. On the other hand, NWs with TB spacing equal to or larger than 12.5 nm show no obvious hardening effects with strain hardening factor *k* very close to 1.0.

## Discussion

**Figure 3:**
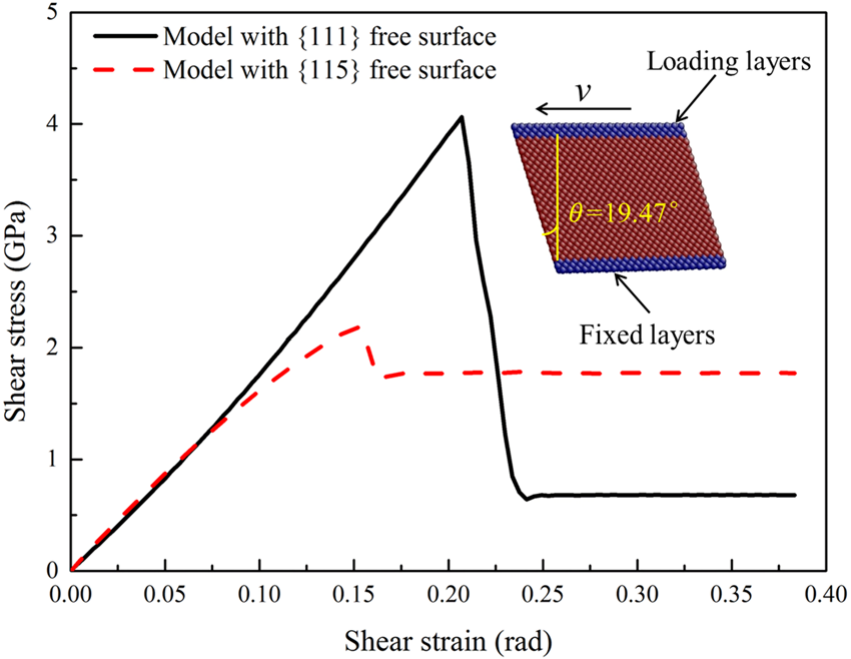
Shearing stress-strain curves of single crystalline models with {111} or {115} free surface.

It is interesting to be found that partial dislocations can only nucleate and slip in the grains with {115} free surface for 19.47° sample. To explain this phenomenon, with using similar method^19^, simulations of shear deformation in single crystalline models with {115} or {111} free surface are carried out. Three layers of atoms at the bottom of the model are fixed and another three layers of atoms at the top of the model move at a speed of 0.12 Å/ps (equivalent to a shearing strain rate of 0.11°/ps), as shown in the inset of Fig. 3. The shearing stress-strain curves of these two models indicate that the critical resolved shear stress (CRSS) is lower in the model with {115} free surface than that with {111} free surface. The difference of CRSS between the two samples mainly stems from the surface energy difference^31^. The surface energy is calculated as 1.4454 J^.^m^−2^ for {115} planes and 1.2385 J^.^m^−2^ for {111} planes (see Supplementary Fig. S4). The difference of CRSS and the surface energy are expected to explain the selectivity of partial dislocations nucleation and slip in the grains with {115} free surface.

**Figure 4:**
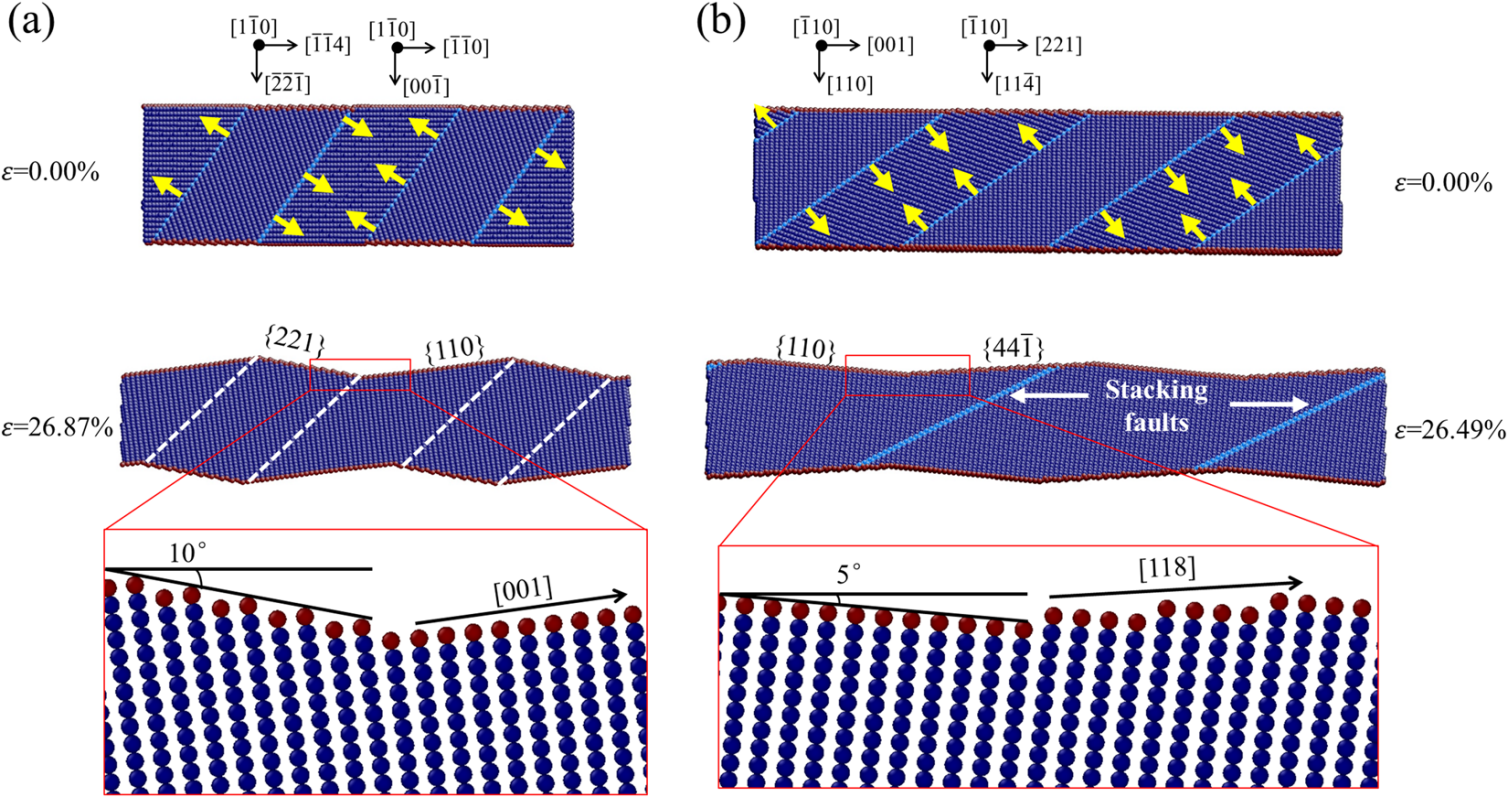
Detwinning and lattice reorientation. **(a)** The originally twinned NW with twin orientation of 35.26° transforms into a single crystalline NW with the disappearance of grains with {001} free surface at the strain of 26.87%. **(b)** For 54.74° sample, CTBs turn into stacking faults as a result of TB migration at the strain of 26.49%. The grains with {114} free surface annihilate at the same time. Yellow arrows and white dashed lines denote the direction of TB migration and the position of TB before disappearing, respectively.

As presented in Fig. 1(c), TB migration in 35.26° and 54.74° samples also shows obvious directionality. To further study the deformation mechanisms of such two samples, details of detwinning and lattice reorientation are given in Fig. 4. For 35.26° sample, TB migration is observed, accompanied by the disappearance of grains with {001} free surface, as illustrated in Fig. 4(a). At the strain of 26.87%, detwinning and lattice reorientation lead to a zigzag-structured single crystalline NW. The new configuration consists of two parts. One is from the original grain with 〈114〉 axis and {221} free surface rotated clockwise by 10 degrees. The other is a newly formed grain with 〈001〉 axis and {110} free surface, as shown in the close-up view. For 54.74° sample, a similar configuration can be observed in Fig. 4(b), which consists of the gain rotated clockwise by 5 degrees from the original one with 〈001〉 axis and {110} free surface and a newly formed grain with 〈118〉 axis and {44$\bar{1}$} free surface. The grain rotation has also been reported in thin gold films and Pt nanograins by *in-situ* experiments^32, 33^. Previous MD simulations and *in-situ* tensile testing have revealed that reorientation from 〈110〉/{111} to 〈001〉/{001} is observed in the tensile loading for Cu^28, 34, 35^, Ni^36^, Au^37^ and Pb^38^ NWs, which is completed through TB migration. In our simulations, the grains with 〈110〉 axis transform into grains with 〈001〉 axis in 35.26° sample and the original grains with 〈001〉 axis are retained in 54.74° sample. Such observations suggest that grains with 〈001〉 axis are preferred upon tensile loading, which could be the reason for the directionality of TB migration.

**Figure 5:**
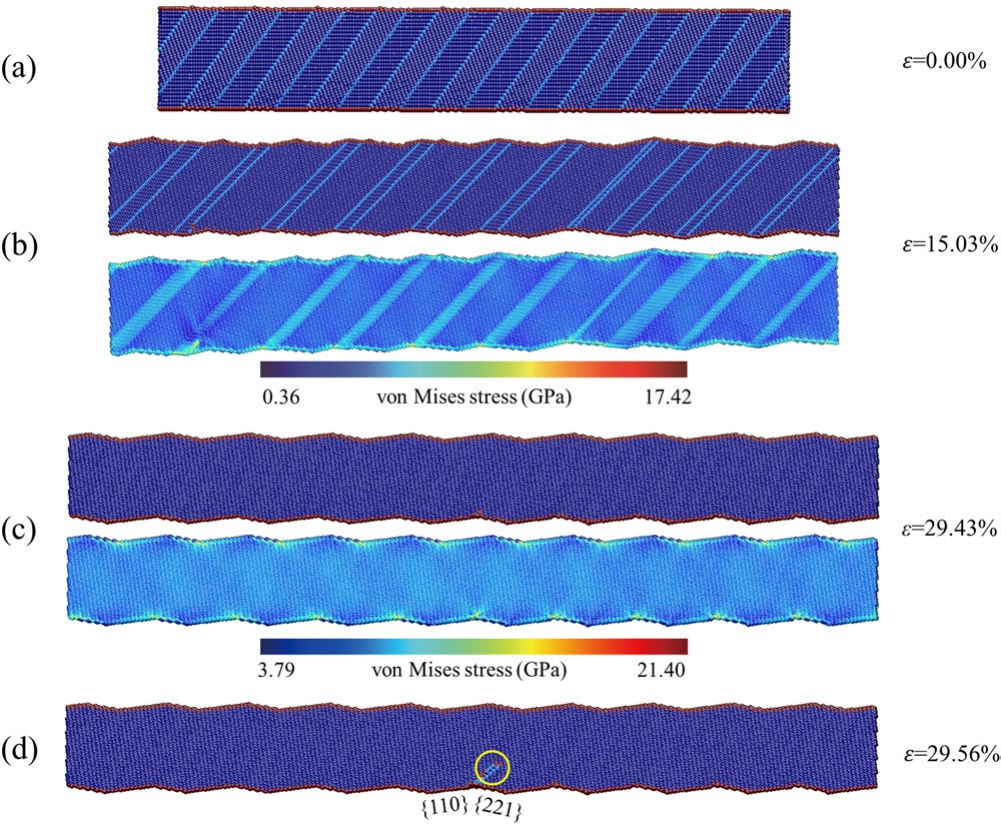
Snapshots of tensile deformation for 35.26° sample with TB spacing *h*_CTB_ = 2.5 nm. **(a)** The original atomic configuration at *ε* = 0.00%. **(b)** The atomic configuration and von Mises stress distribution at the strain of 15.03%. **(c)** The atomic configuration near the peak stress (*ε* = 29.43%). The NW has transformed into a single crystalline NW. **(d)** At *ε* = 29.56%, partial dislocations emit from the junctions of {221} and newly formed {110} free surfaces where stress concentration occurs.

**Figure 6:**
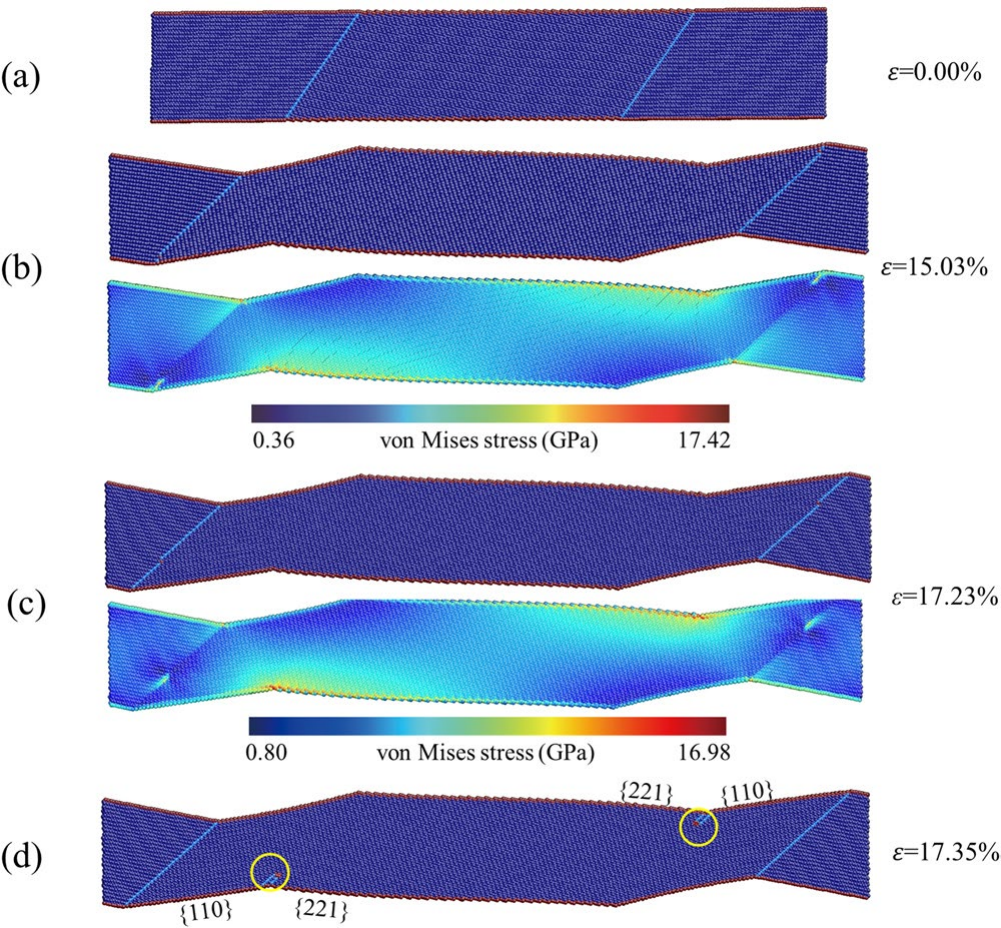
Snapshots of tensile deformation for 35.26° sample with TB spacing *h*_CTB_ = 25.0 nm. **(a)** The original atomic configuration at *ε* = 0.00%. **(b)** The atomic configuration and von Mises stress distribution at the strain of 15.03%. **(c)** The atomic configuration at the end of TB migration (*ε* = 17.23%). **(d)** At *ε* = 17.35%, the NW has not yet transformed into a single crystalline NW and partial dislocations emit from the junctions of {221} and newly formed {110} free surfaces where stress concentration occurs.

As described in the previous section, the average flow stress during TB migration shows size independence on TB spacing, whereas the peak stress increases dramatically with the decrease of TB spacing. Seo *et al*. have derived the expression of twin propagation stress (the average flow stress during the <110>/{111} to <100>/{100} reorientation process) using surface energy differential model^38^. Their results show that the average flow stress during lattice reorientation is equal to *K*/*d*, where *d* is the diameter of the NW and *K* is the lattice reorientation factor, which is associated with the surface energy and the geometry of the free surfaces. In our simulations, the twinned NWs are dimensionally identical and the volume fraction of each grain is 50%. TB migration occurs in each sample by a coinstantaneous reorientation from the original grains with 〈110〉 axis and {001} free surface to the newly formed grains with 〈001〉 axis and {110} free surface. These conditions make the average flow stress theoretically equal for NWs with different TB spacing. To further explore the origin of size dependency in strength of the NWs with titled twins, the snapshots of tensile deformation for 35.26° samples with TB spacing *h*_CTB_ = 2.5 nm and 25.0 nm are presented in Figs 5 and 6. Under the same level of strain, the stress concentration in NWs becomes more significant with TB spacing increasing (Figs 5(b) and 6(b)). As shown in Fig. 5(c), at the strain of 29.43%, the NW has already turned into a single crystalline NW caused by TB migration and undergone a stage of elastic stretching corresponding to the linear increase of stress in the later period of strain hardening (Fig. 2(a)). Von Mises stress contour indicates that stress concentration occurs in the grooves of the free surface. And partial dislocations emit subsequently from the junctions of {221} and newly formed {110} free surfaces, leading to the sharp stress drop (Fig. 5(d)). For the NW with TB spacing *h*_CTB_ = 25.0 nm, at the strain of 17.23%, the free surface is so rough and stress concentration is serious that partial dislocations emit before the NW transforms into a single crystalline NW (Fig. 6). For the 54.74° samples (Supplementary Figs S2 and S3), dislocation slipping intersects with stacking faults retained in the NW with large TB spacing while the one with small TB spacing can transform into defect-free single crystalline NW, which can also be attributed to the surface roughness by the twin reorientation.

**Figure 7:**
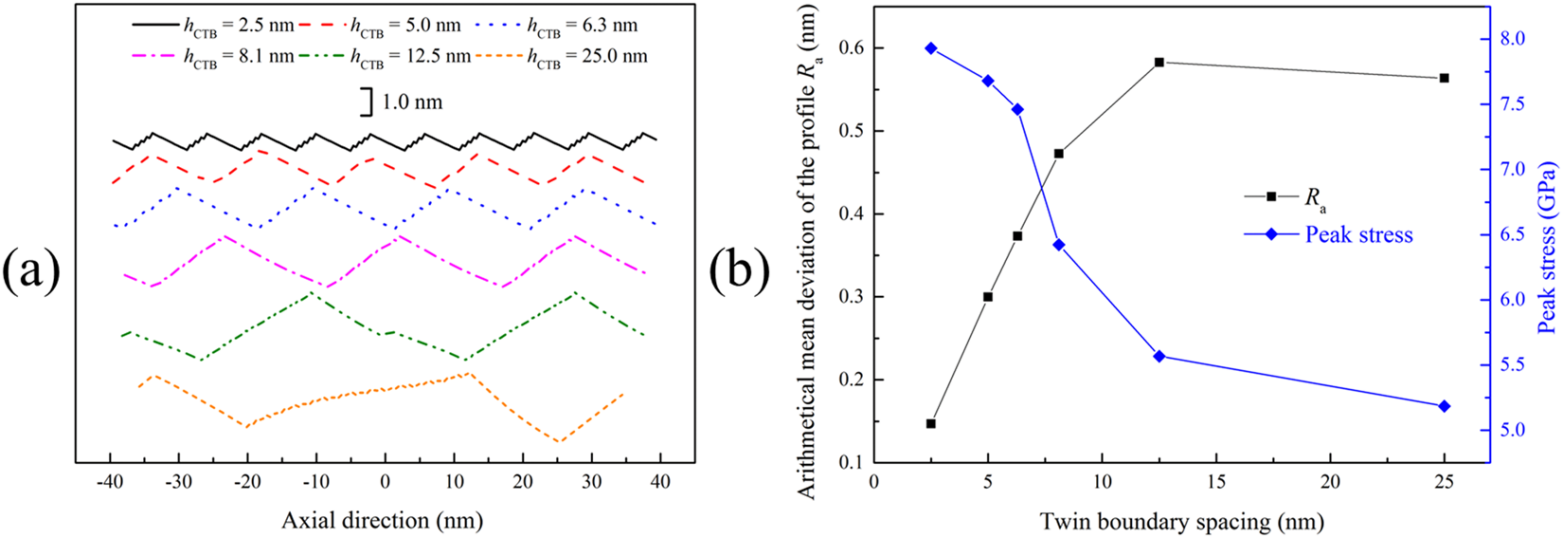
(**a**) The surface profiles of deformed NWs at the strain prior to the maximum flow stress point. **(b)** The arithmetical mean deviation of the profile *R*_*a*_ and the peak stress versus TB spacing.

Based on the above discussion, we may safely draw a conclusion that with the increase of TB spacing, the free surface of the NW during tensile deformation becomes rougher, which is responsible for the decrease of peak stress. To verify this hypothesis, the surface profiles of deformed NWs at the strain prior to the maximum flow stress are shown in Fig. 7(a). It is illustrated that the larger TB spacing is, the more uneven the free surface is. The arithmetical mean deviation of the profile *R*_*a*_ (determined by averaging the absolute value of the offset to the midline of the profile) is adopted as the surface roughness for quantitative characterization. Figure 7(b) shows that *R*_*a*_ increases almost linearly when TB spacing is smaller than 10 nm and then converges to a value (about 0.57 nm), in concert with the variation tendency of peak stress. Essentially, the angle between the {221} and the newly formed {110} free surfaces is constant (about 160.5°). The length of each grain of the twinned NW becomes larger with the increase of TB spacing which results in the increase of surface roughness during lattice reorientation.

## Conclusions

In summary, MD simulations are carried out to investigate twin orientation and TB spacing effects on the mechanical behaviors of Cu NWs with titled twins. There are three types of tensile deformation mechanisms: dislocations slip intersecting with the CTBs (0°, 70.53° and 90° samples), stacking faults parallel to the CTBs formed in favored grains (19.47° sample) and TB migration completed through the propagation of Shockley partial dislocations (35.26° and 54.74° samples). The difference in deformation modes is mainly determined by the combination of Schmid factors, free surface and axial orientation. CRSS and the surface energy take control of the selectivity of partial dislocations nucleation and slip in the favored grains for 19.47° sample. It is proved that grain with 〈001〉 axis is a favored configuration under tensile loading, which could be responsible for the directionality of TB migration in 35.26° and 54.74° samples. The initial yield stress and the average flow stress during TB migration show no obvious size dependence on TB spacing. There is a critical TB spacing *h*_CTB_ = 12.5 nm which divides the yielding mechanisms of NWs into two categories: yield with strain hardening (*h*_CTB_ < 12.5 nm) and yield with nearly constant flow stress (*h*_CTB_ ≥ 12.5 nm). The maximum flow stress increasing with the TB spacing decreasing can be attributed to the surface roughness by lattice reorientation.

## Methods

MD simulations are performed using the large scale atomic molecular dynamics code LAMMPS^39^. We choose the embedded-atom-method (EAM) interatomic potential developed by Y. Mishin *et al*.^40^ to describe the interactions between Cu atoms. In the part of twin orientation effect, Cu NWs with 4 CTBs uniformly distributed are constructed. Each twinned NW has a 10 nm × 10 nm square cross section and the TB spacing is about 6 nm (see Supplementary Fig. S1). The twin orientation *θ* (the angle between the loading axis and the normal of twin plane) can be tunable from 0° to 90° by choosing a specific twin orientation with respect to the loading axis (Here, Z direction is identified as the loading axis). Specifically, in order to maintain dimensional consistency of cross section and TB spacing among twinned NWs with different inclination angles, there are only 2 CTBs at the angle of 90°. The lattice orientations of NWs at 6 inclination angles (0°, 19.47°, 35.26°, 54.74°, 70.53° and 90°) can be found in Supplementary Table S1. NWs with slanted twins at the angle of 35.26° are chosen for research on TB spacing effect. The length of such NWs is about 60 nm and different numbers of twins are created. Periodic boundary condition (PBC) is imposed along the loading axis in order to eliminate the end effects, while the NW is kept free in other directions. The NWs are first relaxed using the conjugate method and then equilibrated at NPT ensemble at 0.1 K for 50 ps to approach zero stress in Z direction. The purpose of maintaining the temperature at 0.1 K in the simulations is to eliminate the effect of thermal oscillation on the observation of dislocations events. Sometimes the plastic deformation mechanism can be changed by the temperature variation, but the twinning mechanism proposed in this paper is found to hold at finite temperatures. Some comparative results of the 3 representative models (0°, 19.47° and 35.26° samples) at the temperature of 0.1 K and 300 K are presented in Supplementary Figs S5–S7. Following equilibration, uniaxial tensile deformation is performed along Z direction with NVT ensemble at a constant strain rate of 5×10^8^ s^−1^. The tensile stress is calculated according to the Virial theorem^41^. In order to capture the defect evolution in NWs, the atoms are colored according to the local crystallinity classification visualized by common neighbor analysis (CNA)^42^. Here, the perfect face centered cubic (FCC), hexagonal close packed (HCP) and non-structured atoms are colored dark blue, light blue and red, respectively. Visualization of the atomistic configurations is performed using ATOMEYE^43^.

## Electronic supplementary material

Supplementary information
