# Supplementary material for: Effects of twin orientation and spacing on the mechanical properties of Cu nanowires
Source: Sci Rep. 2017 Aug 30;7:10056. doi: 10.1038/s41598-017-10934-6 (PMC5577259; doi:10.1038/s41598-017-10934-6)
Supplement: Supplementary file 1 — Supplementary information [file 41598_2017_10934_MOESM1_ESM.pdf]

Supplementary information for "Effects of twin orientation and spacing on the mechanical properties of Cu nanowires"

Zhenyu Yang[a]*, Lingli Zheng[a], Yonghai Yue[b], Zixing Lu[a]

[a]Institute of Solid Mechanics, School of Aeronautic Science and Engineering, Beihang University (BUAA), Beijing 100083, P. R. China

[b]School of Chemistry, Beihang University (BUAA), Beijing 100083, P. R. China

Details for the geometrical model of the NWs with slanted twins

Cu NWs with 4 CTBs uniformly distributed are constructed, as shown in Supplementary Fig. S1. Each twinned NW has a 10 nm×10 nm square cross section and the twin boundary spacing is about 6 nm (denoted as h_{CTB}). The twin orientation θ can be tunable from 0° to 90° by choosing a specific twin orientation with respect to the loading axis (Here, Z direction is identified as the loading axis). Specifically, in order to maintain dimensional consistency of cross section and twin boundary spacing among twinned NWs with different inclination angles, there are only 2 CTBs at the angle of 90°. The lattice orientations of NWs for 6 inclination angles (0°, 19.47°, 35.26°, 54.74°, 70.53° and 90°) are listed in Supplementary Table S1. It can be noticed that there are 3 pairs of complementary angles because of the 90-degrees lattice rotation about Y axis.

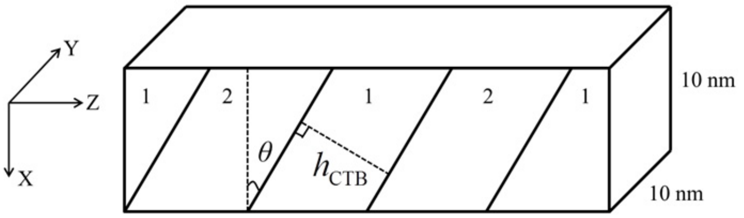

Supplementary Figure 1. **Schematic of Cu NWs containing 4 titled CTBs and two types of grains 1 and 2. The twin orientation θ can be tunable from 0° to 90°.**

* Author to whom correspondence should be addressed. Electronic mail: zyyang@buaa.edu.cn

Supplementary Table 1. The lattice orientations of twinned Cu NWs for 6 inclination angles.

Inclination angle	Grain 1			Grain 2		
	X	Y	Z	X	Y	Z
0°	$[1\bar{1}0]$	$[11\bar{2}]$	$[111]$	$[\bar{1}10]$	$[11\bar{2}]$	$[\bar{1}\bar{1}\bar{1}]$
19.47°	$[1\bar{1}\bar{1}]$	$[110]$	$[1\bar{1}2]$	$[\bar{1}1\bar{5}]$	$[110]$	$[5\bar{5}\bar{2}]$
35.26°	$[00\bar{1}]$	$[1\bar{1}0]$	$[\bar{1}\bar{1}0]$	$[\bar{2}\bar{2}\bar{1}]$	$[1\bar{1}0]$	$[\bar{1}\bar{1}4]$
54.74°	$[110]$	$[\bar{1}10]$	$[001]$	$[11\bar{4}]$	$[\bar{1}10]$	$[221]$
70.53°	$[\bar{1}1\bar{2}]$	$[\bar{1}\bar{1}0]$	$[\bar{1}11]$	$[\bar{5}52]$	$[\bar{1}\bar{1}0]$	$[1\bar{1}5]$
90°	$[\bar{1}\bar{1}\bar{1}]$	$[\bar{1}\bar{1}2]$	$[\bar{1}10]$	$[111]$	$[\bar{1}\bar{1}2]$	$[1\bar{1}0]$

Tensile deformation of Cu NW with twin orientation of 54.74°.

Supplementary Fig. S2 illustrates the plastic deformation of 54.74° sample subjected to axial tension. Twinning partial dislocations nucleate from the junctions of the CTB-free surface at the initial yield strain of 4.71% (Point A). With the loading increasing, partial dislocations propagate on the twin planes at the strain of 5.02% (Point B). At the strain of 5.13% (Point C), the primal dislocations annihilate on the free surface with CTBs moving a distance of one atomic layer and another TB migration begins simultaneously. The two neighboring CTBs turn into stacking faults as a result of TB migration at the strain of 26.49% (Point D). New dislocations nucleate from the junctions of {110} surface and the newly formed free surface with the stress reaching its limit at the strain of 27.63% (Point E). Dislocations slip intersecting with the stacking faults at the strain of 28.02% (Point F). The atomistic configuration at the strain of 64.87% forebodes that the final failure of 54.74° sample is sliding in the TB, which is also observed in the *in-situ* experiments[1].

When the TB spacing reduces to 1.7 nm, the 54.74° sample can totally transform into a single crystalline NW via TB migration, as shown in Supplementary Fig. S3. Previous *in-situ* experiments[2] showed that ultrahigh-density twins were shown to give rise to homogeneous dislocation nucleation. With the TB spacing decreasing, there is

a transition of the plastic deformation in the 54.74° sample.

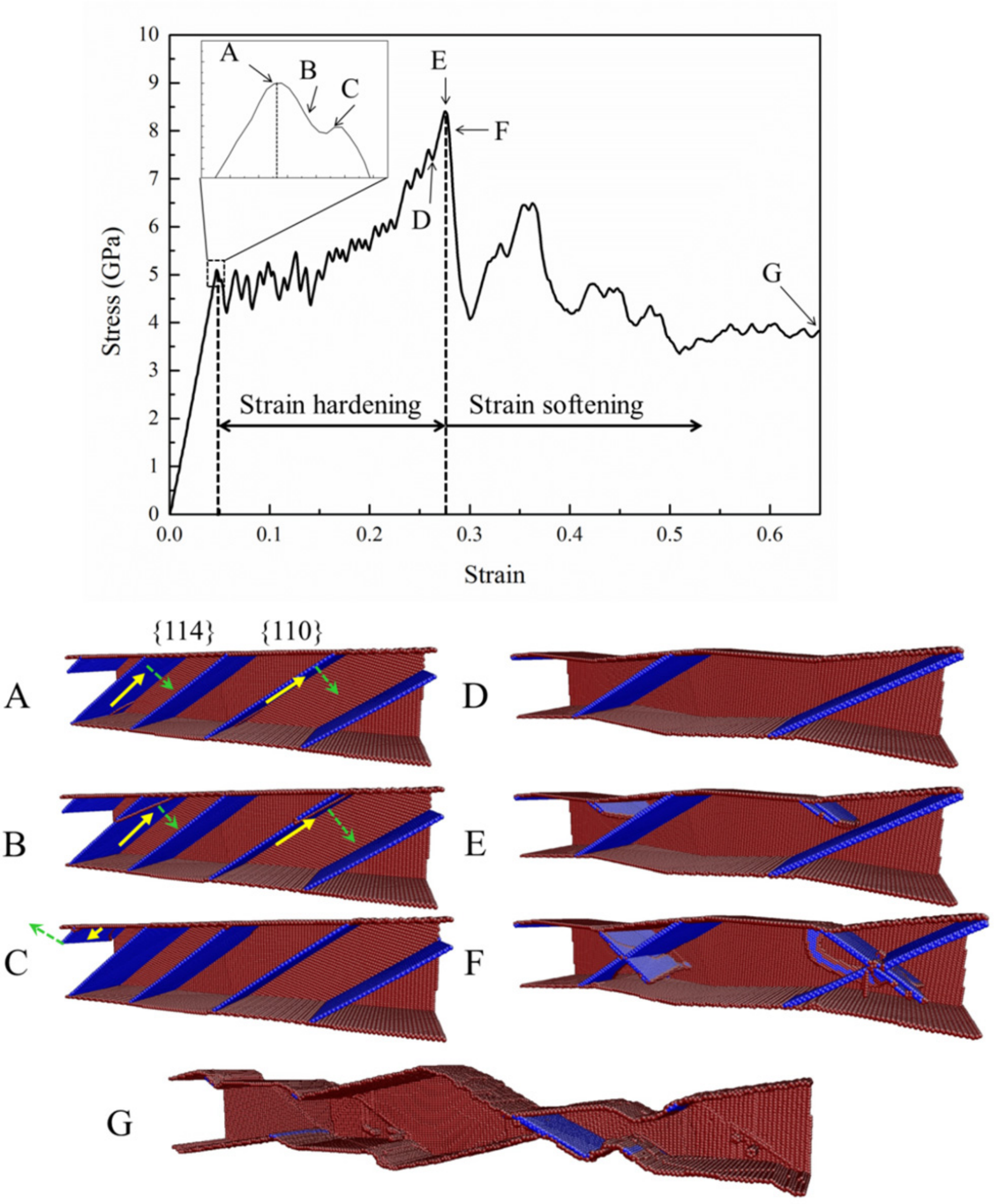

Supplementary Figure 2. Tensile stress-strain curve and plastic deformations for the Cu NW with twin orientation of 54.74° and TB spacing of 6 nm. Yellow arrows and green dashed arrows denote the direction of dislocations slip and TB migration, respectively.

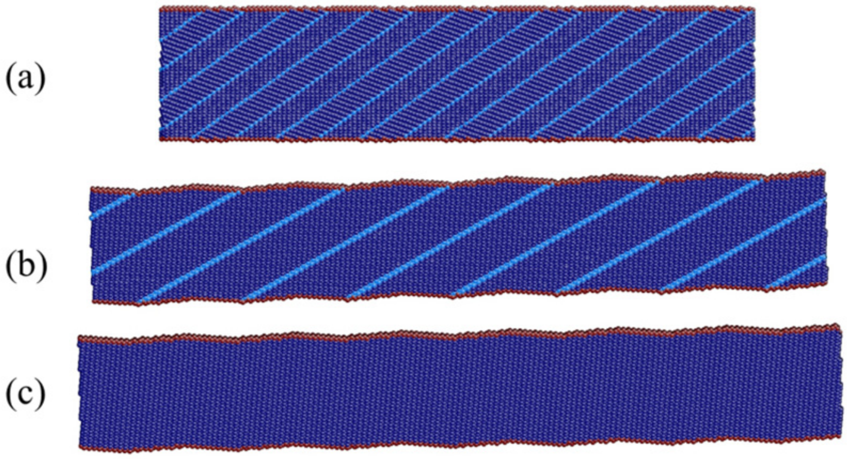

Supplementary Figure 3. Atomistic configurations for the Cu NW with twin orientation of 54.74° and TB spacing of 1.7 nm. (a) ε=0 **(b)** CTBs turn into stacking faults through TB migration at ε=23.61%. **(c)** At ε=27.12%, the stacking faults formed by TB migration completely disappear and the twinned NW transforms into a defect-free single crystalline NW.

Calculation of surface energies

A simple method is adopted to calculate the surface energy (Supplementary Fig. S4). A block with all directions set periodic and the corresponding nanofilm with desired free surface are relaxed at NVT ensemble to reach the equilibrium state. The energy difference (ΔE) between such two models divided by the total free surface area ($2S$) is the surface energy.

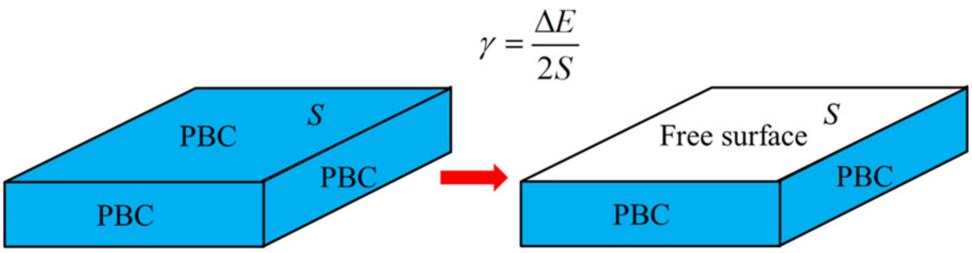

Supplementary Figure 4. The schematic of calculating surface energy

Comparative results at the temperature of 0.1 K and 300 K

The purpose of maintaining the temperature at 0.1 K in the simulations is to eliminate the effect of thermal oscillation on the observation of dislocations events.

Sometimes the plastic deformation mechanism can be changed by the temperature variation, but the twinning mechanism proposed in this paper was found to hold at finite temperatures. Here, some comparative results of the 3 representative models (0°, 19.47° and 35.26° samples) at the temperature of 0.1 K and 300 K are presented in Supplementary Figs. S5-S7. Thermal effect is significant at 300 K with clutter atoms inside the NWs, and the peak stress is lower than that at 0.1 K under the same level of strain, which means both Young's modulus and yield strength decrease with the temperature increasing. However, the plastic deformation mechanisms are similar: (1) dislocations slip intersecting with the CTBs resulting in strain softening at the yield point (0°, 70.53° and 90° samples); (2) the formation and annihilation of stacking faults dominate the plastic deformation process (19.47° sample); (3) TB migration (35.26° and 54.74° samples).

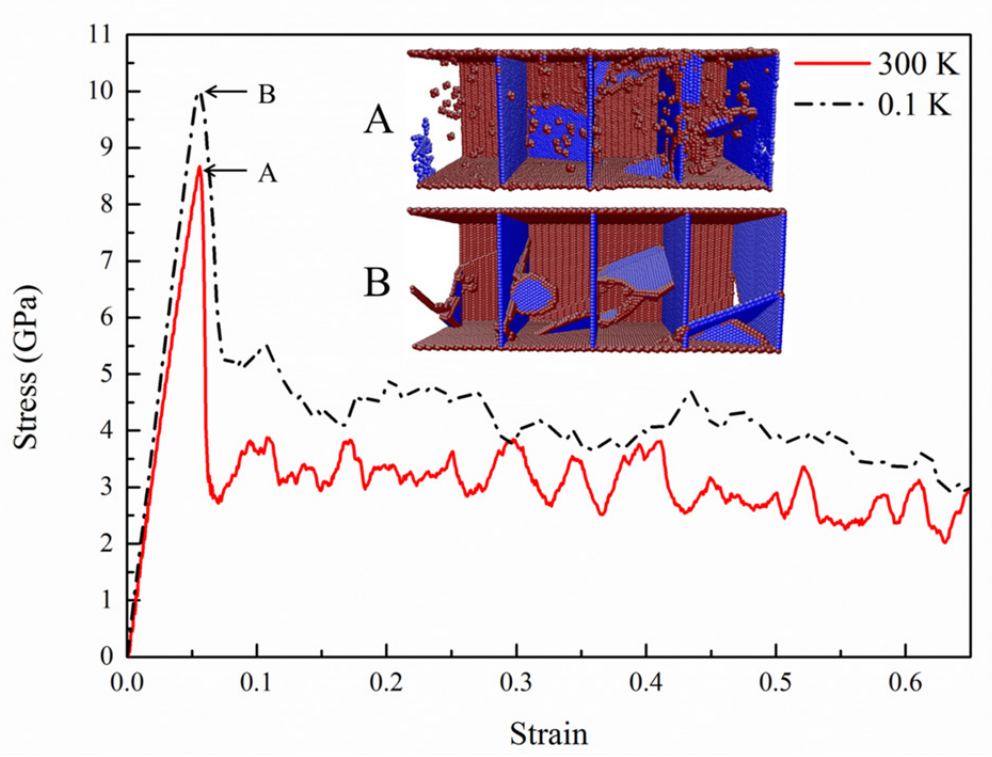

Supplementary Figure 5. Tensile stress-strain curves and plastic deformations for Cu NW with twin orientation of 0° at 0.1 K and 300 K. Dislocations slip in the planes inclined to the CTBs at the initial yield strain. The front surface atoms and the perfect FCC atoms are removed for clarity.

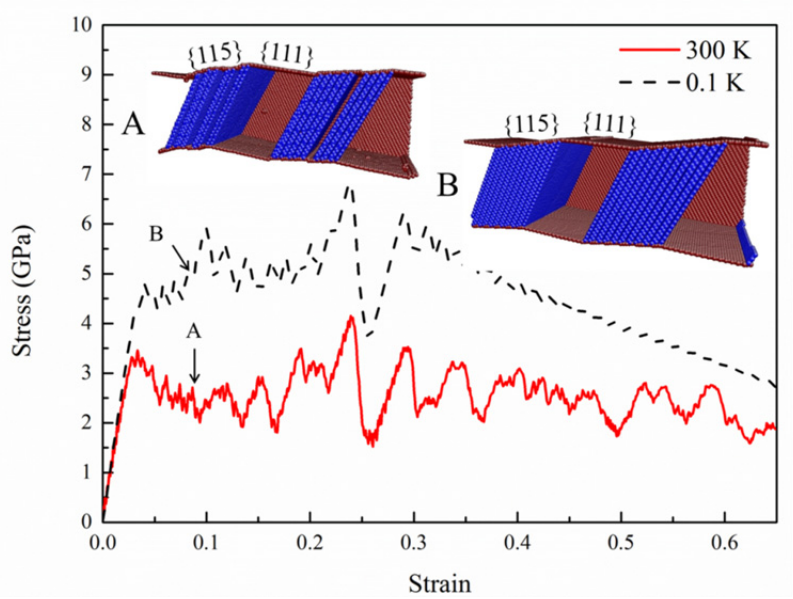

Supplementary Figure 6. Tensile stress-strain curves and plastic deformations for Cu NW with twin orientation of 19.47° at 0.1 K and 300 K. The formation and annihilation of stacking faults in the grains with {115} free surface dominate the plastic deformation process. The front surface atoms and the perfect FCC atoms are removed for clarity.

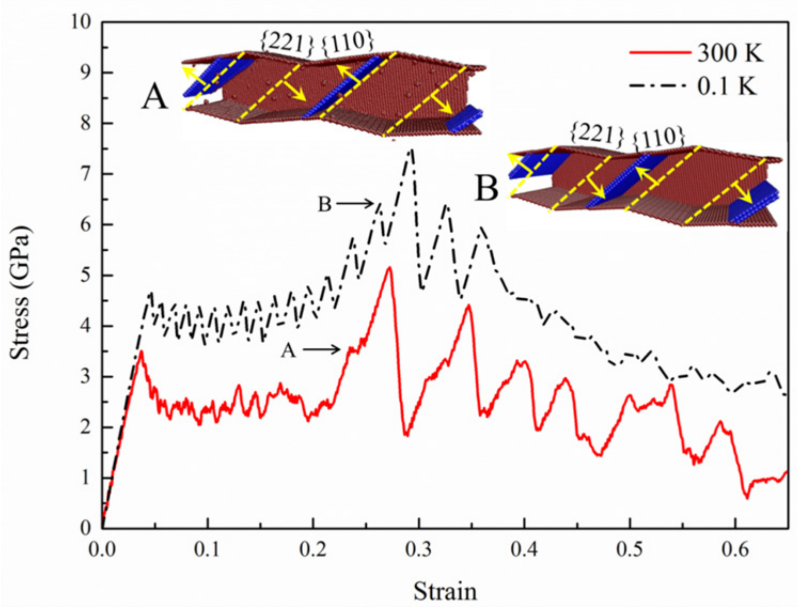

Supplementary Figure 7. Tensile stress-strain curves and plastic deformations for Cu NW with twin orientation of 35.26° at 0.1 K and 300 K. TB migration is observed in the process of strain hardening. The arrows and dashed lines denote the direction of TB migration and the initial position of TB, respectively. The front surface atoms and the perfect FCC atoms are removed for clarity.

References

1. Yue, Y. *et al.*, In situ observation of twin boundary sliding in single crystalline Cu nanowires. *Small* 1604296; 10.1002/smll.201604296 (2017).

2. Wang, J. *et al.*, Near-ideal theoretical strength in gold nanowires containing angstrom scale twins. *Nature Communications*. **4** 1742 (2013).
